# Does honey have any salutary effect against streptozotocin - induced diabetes in rats?

**DOI:** 10.1186/s40200-016-0278-y

**Published:** 2017-01-24

**Authors:** Zakariya M. Al Aamri, Badreldin H. Ali

**Affiliations:** 1 College of Medicine & Health Sciences, Sultan Qaboos University, Al - Khoud, Oman; 2 Department of Pharmacology, College of Medicine & Health Sciences, Sultan Qaboos University, AL-Koud, Oman

**Keywords:** Diabetes, Honey, Insulin, Blood glucose, Body weight, Leptin

## Abstract

**Background:**

Diabetes is a global, growing and costly public health problem. In the literature, there are conflicting reports on the effect of consumption of bee honey on diabetes. We assessed the possible effect of a commercially available bee honey (given orally by gavage at doses of 1 g/kg/day for 4 weeks) on the blood concentrations of glucose, insulin and leptin and body weight of rats with streptozotocin-induced diabetes.

**Methods:**

Thirty-six rats were allocated randomly into six groups equally and treated for 4 weeks as follows: Group.1: non-diabetic rats given distilled water, group.2: non-diabetic rats given honey (1 g/kg), group.3: Diabetic rats given distilled water, group.4: Diabetic rats given honey, group.5: Diabetic rats given insulin (10 IU/kg), and group.6: Diabetic rats given combination of insulin (10 IU/kg) with honey (1 g/kg). The body weight, blood glucose, insulin and leptin concentrations of each rat were measured.

**Results:**

Honey treatment did not significantly affect the glucose, leptin and insulin concentrations of diabetic rats. It did not significantly affect the excessive water intake or urinary output in diabetic rats when compared to the insulin-treated groups. Neither honey nor insulin improved body weight in diabetic rats.

**Conclusion:**

Contrary to the reports of a salutary effect of honey in diabetic humans and rodents, our results showed that consumption of honey caused no significant changes in body weight, or glucose and insulin concentrations. However, further studies with different doses and durations of treatment are warranted.

## Background

Diabetes is an abnormal metabolic state that results in hyperglycemia due to either insulin deficiency or abnormal response to insulin, and is of two types: type I, which is caused by severe deficiency in insulin synthesis, and type II diabetes that is complicated with insulin resistance and/or insulin production deficiency [1]. In 2014, it has been estimated that there are about 387 million diabetic patients worldwide [2]. Consequently, studies conducted to find out an ideal remedy against diabetes are needed, and bee honey has been suggested as one of these [3, 4].

Bees honey from bees (*Apis mellifera*) is produced from nectar and it consists of at least 181components including fructose (37.5%), glucose (30.6%), sucrose (1.62%), maltose (2.7%), water (17.2%), vitamins, minerals, enzymes, acids such as flavonoids, phenolic acids and other components [3–9]. Honey’s constituents differ slightly depending on the botanical species [10, 11].

In folk medicine, honey is used in different parts of the world for a number of unrelated diseases and conditions [5]. Furthermore, it is known to possess antioxidant, anti-inflammatory and antimicrobial properties [4, 11–13]. Moreover, it has been reported that honey has effects against hyperglycemia in diabetic patients and against complications of diabetes. Its anti-hyperglycemic effects may relate to its fructose content and antioxidant properties [4, 6]. Fructose is a monosaccharide sugar that has slow absorption rate and its intake may lead to delaying the digestion and elongating the gastric emptying. Therefore, the fructose was suggested to contribute to the anti-hyperglycemic effects of the honey. Some studies showed that fructose increases hepatic uptake of glucose resulting in decreasing blood glucose [4]. The antioxidant effects of honey may be due to compounds such as flavonoids and phenolic acids [5, 14–16]. These antioxidants activities of honey may improve oxidative stress in β-cells in the pancreas, and this may lead to promoting insulin secretion. In addition, honey has been found to reduce insulin resistance in type II diabetic patients [17].

In literature, many parameters were used to investigate glycemic control of diabetic patients/animals including blood glucose, body weight, HbA1c and others. Moreover, many other parameters (eg insulin and leptin) were suggested to be used as indicators or predictors for diabetes status or its glycemic control. There is a controversy regarding the association between leptin levels & diabetes. However, it has been reported that blood leptin levels may be used as a predictor for glycemic control [18]. Moreover, Japanese researchers investigated the possible therapeutic effects of leptin in congenital hyperleptinemic diabetic mice models where it showed a salutary effect [19]. Many studies were conducted to find out the effect of honey on diabetic rats in which they investigated blood insulin level and their results were variable [20–22].

In literature, there is a controversy regarding the antihyperglycemic effects of honey on diabetes. Therefore, our study aimed to assess the effect of a commercially available bee honey (1 g/kg/day for 4 weeks) on the concentrations of blood glucose, insulin and leptin and body weight in normal rats and rats with streptozotocin-induced diabetes.

## Methods

### Animal models

This prospective randomized controlled study was carried at Sultan Qaboos University in Muscat, Oman between January 2013 and July 2014. The use of rats and all the experimental procedures were approved by the Animal Ethical Committee at Sultan Qaboos University (SQU/ACE/2012-13/4). Thirty six female Sprague Dawley rats (250–350 g) were obtained from Small Animal House of Sultan Qaboos University, Muscat, Oman and were kept under standard conditions (a temperature of 22 ± 2 °C, relative humidity of about 60%, with a 12 h light–dark cycle (lights on 6:00), and (unless otherwise mentioned) given free access to standard pellet chow diet containing 0.85% phosphorus, 1.12% calcium, 0.35% magnesium, 25.3% crude protein and 2.5 IU/g vitamin D3 (Oman Flour Mills, Muscat, Oman) and water. Ethical clearance was obtained from our University Animal Ethics Committee and all procedures involving animals and their care were carried out in accordance with international laws and policies (EEC Council directives 86/609, OJL 358, 1 December, 12, 1987; NIH Guide for the Care and Use of Laboratory Animals, NIH Publications No. 85-23, 1985).

### Induction of diabetes

Diabetes was induced after 18 h fasting by intraperitoneal (i.p.) injection of streptozotocin (STZ) at a dose of 50 mg/kg, dissolved in 0.1 M of a citrate buffer (pH 4.5) [23]. Other groups of rats were injected with citrate buffer. Two days thereafter, the blood glucose (BG) concentration was checked using a OneTouch® UltraMini® Meter (LifeScan, Milpitas, CA, USA) using a drop of blood obtained by pricking tip tail. Rats with blood glucose concentrations ≥ 14 mmol/L were considered diabetic [12]. Treatments were started 3 weeks after STZ or citrate buffer injections. Body weight was measured daily, fasting blood glucose concentration was measured weekly, and blood insulin & leptin concentrations were measured once at the end of the experiment.

### Experimental design

The animals were allocated randomly into six equal groups and treated for 4 weeks as follows: Group1 (C): non-diabetic and were treated via oral gavage with distilled water (0.5 ml/rat) via oral gavage once daily; Group 2 (H): non-diabetic and were given honey (1 g/kg body weight) via oral gavage once daily [12, 20, 24]; Group 3 (D): diabetic and were treated with distilled water (0.5 ml/rat) via oral gavage once daily; Group 4 (DH): diabetic and were treated with honey (1 g/kg body weight) via oral gavage once daily; Group 5 (DI): diabetic and were treated with insulin (10 IU/Kg body weight) subcutaneously once daily [25]; and Group 6 (DIH): diabetic and were treated with honey (1 g/kg body weight) and insulin (10 IU/Kg body weight) once daily.

After 4 weeks of treatment, rats were placed in metabolic cages to measure water intake & collect 24-h urine. Fasting Blood glucose concentration was measured and rats were then anesthetized (using ketamine (75 mg/kg) and xylazine (5 mg/kg) intraperitoneally) and blood (4 ml) collected from the inferior vena cava. Blood samples were centrifuged (900 g for 15 min at 4 °C) and plasma collected was frozen for subsequent measurement of leptin and insulin concentrations.

Commercially available honey (Golden Glory, Australia) was bought from a local market in Muscat. Honey (1 g/kg/day) was diluted with distilled water before given it to the rats by gavage. As well as, Insulin (10 IU/Kg body weight) was diluted with 0.90% NaCl just before injecting.

### Biochemical analysis

Collected plasma samples were analyzed using ELISA test for both insulin (EIA Kit, Gayman Chemical Company, Michigan, USA) and leptin (EIA Kit, Gayman Chemical Company, Michigan, USA).

### Statistical analysis

Statistical analysis was conducted by the GraphPad Prism 6.0 (GraphPad Software, San Diego, CA, USA). Each group consisted of six rats. All data are given as means ± S.E.M. Group means were compared with an analysis of variance (ANOVA; One-Way) followed by Tukey’s multiple comparison test. Values of *P* < 0.05 were considered significant.

## Results

**Fig. 1:**
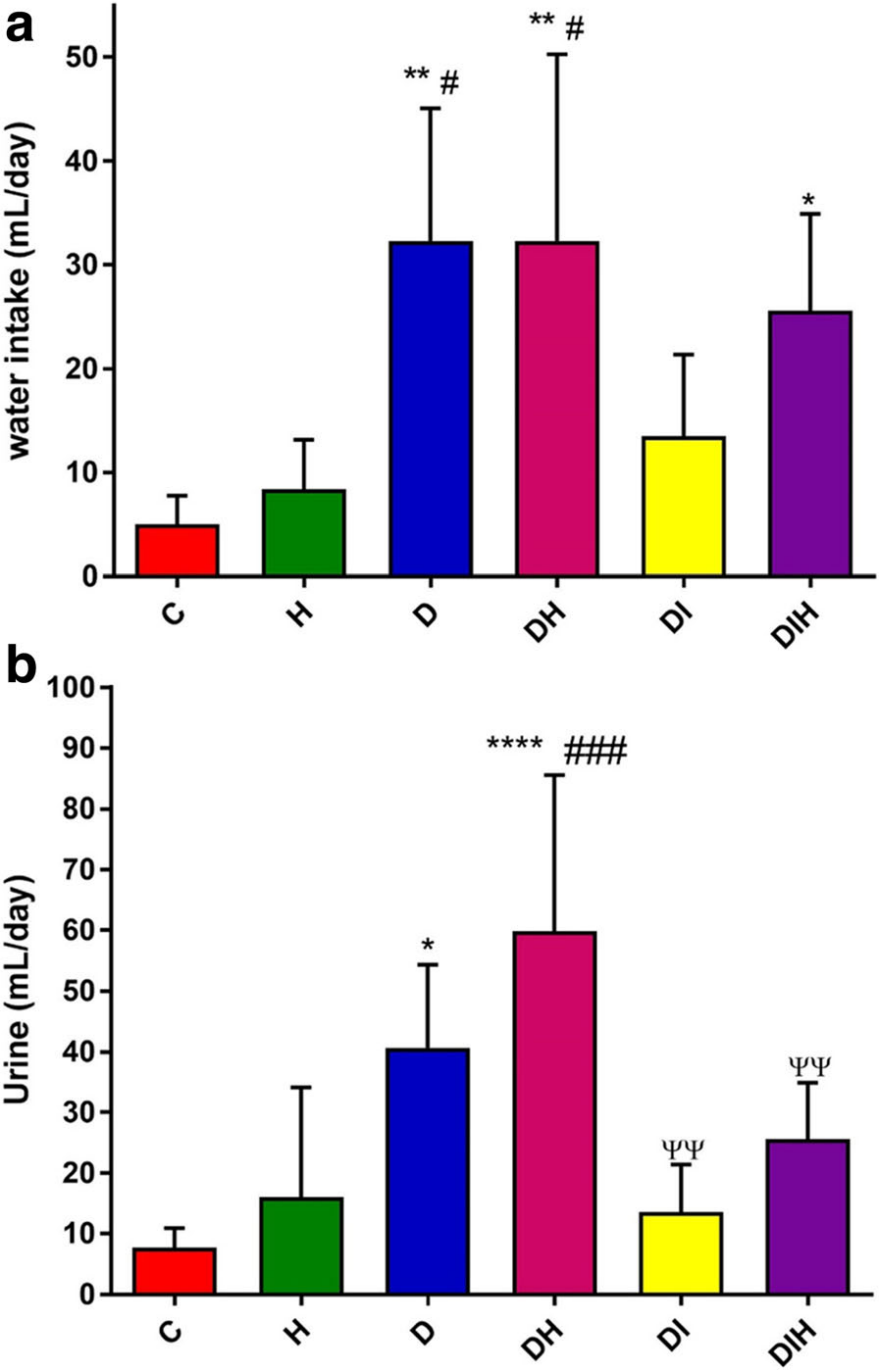
Effect of honey on (**a**) water intake and (**b**) urine output in normal and diabetic rats. Data are expressed as mean ± SEM. Each group consisted of four to six rats. Values are significant at * *p* < 0.05, ** *p* < 0.01, **** *p* < 0.0001 compared to C; # *p* < 0.05, ### *p* < 0.001 compared to H; ^ΨΨ^ *p* < 0.01 compared to DH

Figure 1 summarizes the results of 24 h water intake and urine output of six different groups of rats. The diabetic control animals had significantly (*p < 0.01*) increased water intake compared to non-diabetic control rats. Commercial honey significantly increased water intake in both diabetic group which given honey (*p < 0.01*) and diabetic group that treated with insulin in combination with honey (*p < 0.05*) compared to non-diabetic control rats. However, water intake of diabetic rats that received honey showed no significant difference compared to diabetic control rats. The diabetic control group and diabetic that treated with honey had significantly (*p < 0.05*) higher water intake compared to non-diabetic that received honey.

Urine output of the diabetic control group was significantly (*p < 0.05*) higher than non-diabetic control. The diabetic group that treated with honey alone showed significant increase in urine output compared to non-diabetic control (*p < 0.0001*) and non-diabetic that received honey (*p < 0.001*). Moreover, diabetic with insulin and diabetic that treated with insulin in combination with honey showed significant decrease in urine output compared to diabetic with honey (*p < 0.01*).

Table 1 shows the effects of 4 weeks treatment with honey or insulin, or their combination on body weight of treated rats. There was initially no significant difference in the body weight among all the groups. After induction of diabetes, the groups that injected with streptozotocin had significantly lower body weight compared to the two non-diabetic groups. After starting the treatment of animals, neither honey nor insulin showed a significant improvement in body weight of diabetic rats compared to non-diabetic rats. However, the animals that treated with insulin or a combination of insulin and honey had significantly higher body weight in the fourth week of treatment compared to diabetic group that received honey alone (*p* < 0.01). Along the period of treatment, there was no significant difference between the diabetic group that given honey and the diabetic control group.

**Table 1:** The effect of honey on body weight of normal and diabetic rats

Group	Control	Diabetic	Honey	Diabetic + Honey	Diabetic + Insulin	Diabetic + Insulin + Honey
A week before treatment	302.00 ± 4.95	241.17 ± 12.08 ** ^##^	303.00 ± 13.92	233.17 ± 12.32 ** ^# #^	242.00 ± 10.61* ^#^	261.60 ± 6.86
Weeks after treatment						
1st	307.50 ± 5.80	230.00 ± 13.68*** ^##^	300.60 ± 13.71	220.00 ± 12.25**** ^###^	241.25 ± 16.18** ^#^	252.20 ± 6.15*
2nd	328.50 ± 6.16	260.00 ± 14.76** ^#^	322.00 ± 15.43	238.17 ± 14.43*** ^##^	252.25 ± 16.62** ^#^	251.20 ± 5.44** ^##^
3rd	327.83 ± 6.80	239.17 ± 14.08**** ^###^	323.60 ± 13.82	224.67 ± 8.83**** ^####^	253.50 ± 16.81** ^##^	251.00 ± 7.12** ^##^
4th	324.83 ± 6.16	202.33 ± 14.44 *** ^####^	324.00 ± 12.35	182.33 ± 14.37 *** ^###^	256.25 ± 17.72 * ^#^^ΨΨ^	250.60 ± 6.59 ** ^##^^ΨΨ^

Data are expressed as mean ± SEM (*n* = 4–6 rats). Values are significant at *p < 0.05, **p < 0.01, ***p < 0.001, ****p < 0.0001 compared to C; #*p* < 0.05, ##*p* < 0.01, ###*p* < 0.001, ####*p* < 0.0001 compared to H;^ΨΨ^ *p* < 0.01 compared to DH

(Location: can be added after the second page of the results section)

Table 2 summarizes the effects of 4 weeks treatment of honey and insulin or their combination on fasting serum blood glucose concentration of six different groups of rats. Before injecting the rats with streptozotocin, there was no significant difference in fasting blood glucose concentration among the six groups of the animals. The rats that treated with streptozotocin showed a significant increase in blood glucose concentration in the weeks before the treatment compared to both non-diabetic control group (4.25 ± 0.25) & non-diabetic treated with honey group (4.98 ± 0.22). The diabetic animals that received honey had no significant increase in the blood glucose concentration compared to diabetic control rats. However, diabetic control rats & diabetic rats that given commercial honey had significantly higher blood glucose concentration compared to the two non-diabetic groups along the duration of treatment. Along the 4 weeks of treatment, the rats that treated with insulin showed no significant difference in fasting blood glucose concentration (11.65 ± 2.43, 5.95 ± 0.85, 13.78 ± 4.46, & 8.33 ± 2.15 respectively) compared to non-diabetic control group (5.13 ± 0.24, 4.02 ± 0.10, 4.00 ± 0.22, & 4.82 ± 0.21 respectively) & non-diabetic group that given honey (5.00 ± 0.22, 4.16 ± 0.09, 3.88 ± 0.11, & 4.90 ± 0.16 respectively). In contrast, there was a significant difference between diabetic rats that given insulin on one side and the diabetic control or the diabetic that treated with honey on the other side in most of the weeks of the treatment. The diabetic group that received the combination of honey and insulin had no significant increase in blood glucose concentration compared to the two non-diabetic groups among second, third and fourth week of treatment.

**Table 2:** The effect of honey on blood glucose concentration in the blood of normal and diabetic rats

Group	Control	Diabetic	Honey	Diabetic + Honey	Diabetic + Insulin	Diabetic + Insulin + Honey
A week before treatment	4.25 ± 0.25	13.07 ± 2.48	4.98 ± 0.22	19.60 ± 3.57 ** ^##^	17.60 ± 3.25 * ^#^	25.74 ± 2.28 **** ^$^^####^
Weeks after treatment						
1st	5.13 ± 0.24	23.85 ± 0.83 **** ^###^	5.00 ± 0.22	22.72 ± 3.90 *** ^###^	11.65 ± 2.43 ^$^	17.30 ± 4.00 * ^#^
2nd	4.02 ± 0.10	14.20 ± 3.55	4.16 ± 0.09	15.08 ± 2.86	5.95 ± 0.85	13.80 ± 4.52
3rd	4.00 ± 0.22	19.05 ± 3.52 ** ^##^	3.88 ± 0.11	26.37 ± 2.35 **** ^####^	13.78 ± 4.46 ^Ψ^	15.40 ± 3.59
4th	4.82 ± 0.21	21.55 ± 2.97 *** ^##^	4.90 ± 0.16	22.52 ± 3.72 *** ^###^	8.33 ± 2.15 ^$, Ψ^	11.48 ± 2.77 ^Ψ^

Data are expressed as mean ± SEM (*n* = 4–6 rats). Values are significant at *p < 0.05, **p < 0.01, ***p < 0.001, ****p < 0.0001 compared to C; ^#^ *p* < 0.05, ^##^ *p* < 0.01, ^###^ *p* < 0.001, ^####^ *p* < 0.0001 compared to H; ^$^ *p* < 0.05 compared to D; ^Ψ^ *p* < 0.05 compared to DH

(Location:can be added in the page after that of Table 1)

**Fig. 2:**
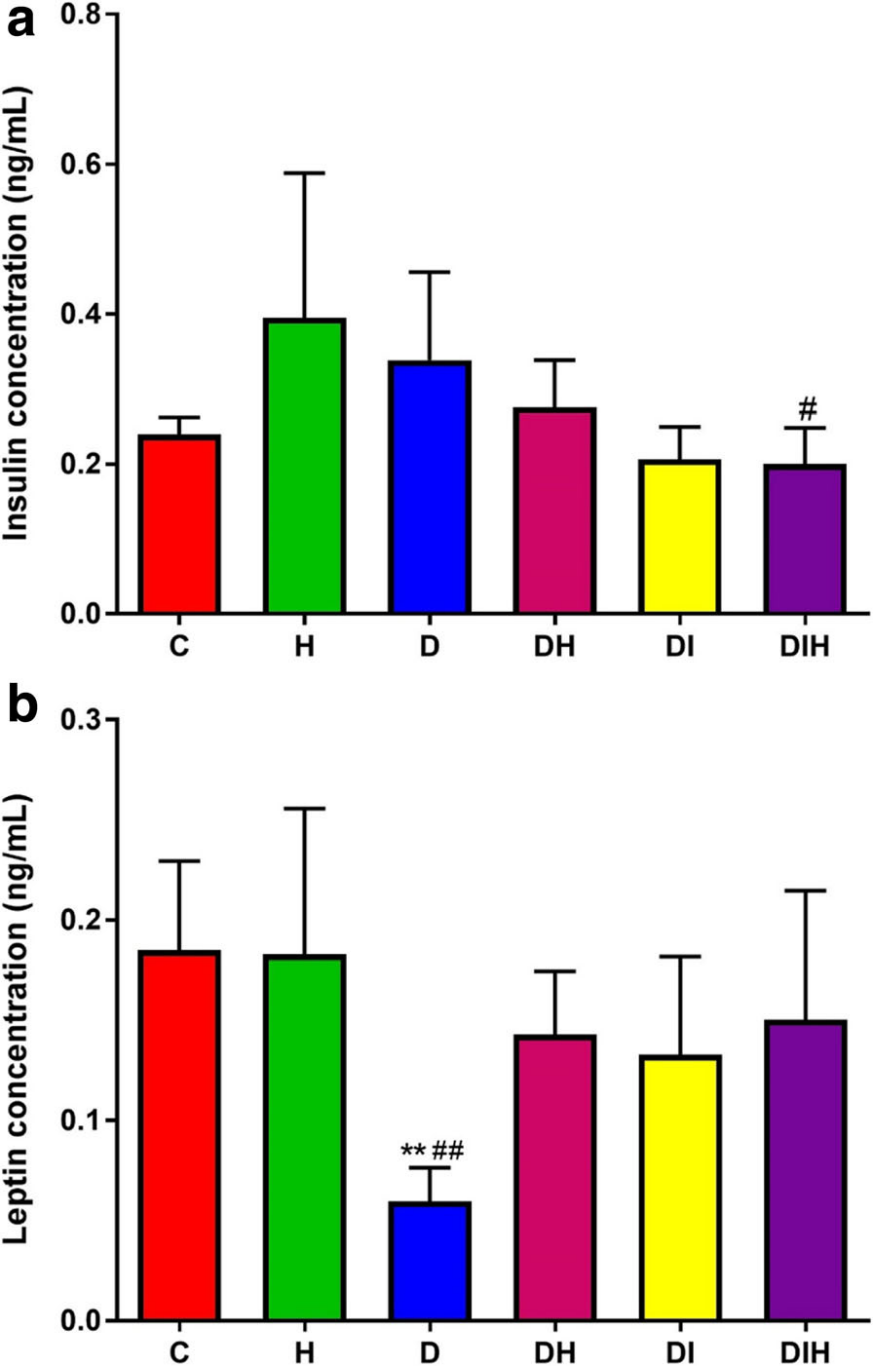
The effect of honey on blood (**a**) insulin & (**b**) leptin concentrations in normal and diabetic rats. Data are expressed as mean and SEM. Each group consisted of four to six rats. Values are significant at ** *p* < 0.01compared to C; # *p* < 0.05, # # *p* < 0.01compared to H

Figure 2 shows the effects of 4 weeks treatment with honey or insulin, or their combination on blood (a) insulin & (b) leptin concentrations. Overall, honey treatment did not show any significant change in blood insulin concentration among all groups of rats. However, diabetic rats treated with honey in combination with insulin showed significant decrease (*p < 0.05*) in blood insulin concentration compared to non-diabetic that received honey as shown in graph (a) in Fig. 2.

The diabetic control group had significantly (*p < 0.01*) lower blood leptin concentration compared to non-diabetic control rats and non-diabetic group that received honey alone. There is no significant difference between non-diabetic groups and diabetic groups that received honey, insulin or their combination (ie honey improved leptin blood concentration).

## Discussion

In the current study, we found that honey treatment did not significantly prevent excessive water intake and urinary frequency in diabetic rats. However, in rats treated with insulin, there were improvements in these two physiological parameters. As far as we are aware, there are no previously reported data on the effects of honey and insulin (given together) on water intake and urine output.

As expected, significant decrease in body weight was observed in STZ-induced diabetic rats, and this may be because of connective tissue degradation due to the surge lysosomal activity [26]. Our study showed that neither honey nor insulin improved the loss in body weight induced by diabetes when compared to non-diabetic rats. In contrast, some previous studies had reported that honey has a beneficial effect in increasing the body weight of the diabetic humans and rats probably due to the fructose component of honey with its purported antihyperglycemic effects [12, 20]. Diabetic rats that were treated with insulin showed body weight gain and this may be due to the effect of insulin in decreasing energy loss via urine [25].

Unlike our present results, some authors in Malaysia [12, 20] reported that the honey they used in their experiments was effective in ameliorating streptozotocin - induced diabetes in rats because honey has anti-oxidant properties against streptozotocin – induced oxidative stress, which leads to β cells destruction. Fructose in the honey may also slows the absorption rate which may lead to delaying the digestion and elongating the gastric emptying and its ability to increase hepatic uptake of glucose resulting in decreasing blood glucose [4]. The antioxidant effects of honey may be attributed to its flavonoids and phenolic acids contents, which decrease the free radicals in β-cells in the pancreas, and this may lead to promoting insulin secretion [5, 14–16]. However, Bahrami and his colleagues reported that honey had no significant difference in blood glucose concentration of diabetic patients with type 2 diabetes. When honey used to treat diabetic rats, they showed no improvement in blood glucose concentration [27].

It is not clear why we could not reproduce the positive results of those who claim honey is beneficial in diabetic rats [12, 20]. The discrepancy may be due to the type of honey used in both experiments. It is known that the composition of honey depends on its floral origin, or to other unknown factors [10, 11].

Plasma insulin concentration is usually significantly reduced in rats with streptozotocin – induced diabetes [28]. However, we found that there is no significant difference in blood insulin concentration among all groups except for diabetic rats given honey in combination with insulin and non-diabetic group given honey. Many studies were conducted to find out the effect of honey on diabetic rats and their results were at variance. Some authors found that honey increases the concentration of insulin concentration but the others showed that honey had no effect on blood insulin concentration [20–22]. Our results seem to insignificantly inceases the plasma concnetrations of insulin.

Leptin is a protein hormone mainly produced by adipocytes and plays a vital role in the regulation of energy intake and expenditure, and is positively associated with body mass index (BMI) and the absolute fat mass, as well as glycemic control [29]. The recent study showed that honey increased leptin blood concentration of diabetic rats given honey, insulin or their combination compared to diabetic control group. Many studies were conducted to investigate the association between leptin and diabetes and their results were contradicted. Some studies showed that there is a direct association between leptin and diabetes development [30–33]. On the other hand, other studies found that there is no association between leptin and diabetes [34, 35]. A randomized control trial on rats showed that the rats treated with honey have lower blood leptin concentration compared to those treated with sucrose [22].

The possible limitations of this experiment are the small number of rats in each group and the use of a single type of honey at a single dose. Further experiments using different types of honey at different doses against streptozotocin-induced diabetes are warranted.

## Conclusion

Based on our results, the present preliminary experiment suggests that the use of honey as a possible treatment in streptozotocin-induced diabetes in rats is not beneficial.

## Abbreviations

BG: Blood glucose
i.p.: Intraperitoneal
IU: International unit
STZ: Streptozotocin
